# Identifying Mixture Components From Large-Scale Keystroke Log Data

**DOI:** 10.3389/fpsyg.2021.628660

**Published:** 2021-07-29

**Authors:** Tingxuan Li

**Affiliations:** School of Education, Shanghai Jiao Tong University, Shanghai, China

**Keywords:** computer-based assessment, keystroke log data, cognitive, writing, finite mixture model (FMM)

## Abstract

In a computer-based writing assessment, massive keystroke log data can provide real-time information on students’ writing behaviors during text production. This research aims to quantify the writing process from a cognitive standpoint. The hope is that the quantification may contribute to establish a writing profile for each student to represent a student’s learning status. Such profiles may contain richer information to influence the ongoing and future writing instruction. Educational Testing Service (ETS) administered the assessment and collected a large sample of student essays. The sample used in this study contains nearly 1,000 essays collected across 24 schools in 18 U.S. states. Using a mixture of lognormal models, the main findings show that the estimated parameters on pause data are meaningful and interpretable with low-to-high cognitive processes. These findings are also consistent across two writing genres. Moreover, the mixture model captures aspects of the writing process not examined otherwise: (1) for some students, the model comparison criterion favored the three-component model, whereas for other students, the criterion favored the four-component model; and (2) students with low human scores have a wide range of values on the mixing proportion parameter, whereas students with higher scores do not possess this pattern.

## Introduction

Modern technology has made large-scale digital data available in many disciplines (Donoho, 2017). Making sense of these data sources requires new perspectives, coupled with possession of the substantive knowledge in a particular discipline (Chen et al., 2020). In educational research, scholars have examined the possibilities for massive digital data to enrich teaching, learning, and assessment of those activities (Romero and Ventura, 2020). For example, data extracted from short texts on social media (e.g., Twitter) were used as predictors of students’ academic performance (Smirnov, 2020). Khosravi et al. (2017) explored students’ knowledge gap and interests in online collaborative learning environment.

In computer-based assessment platforms, *process data* have received substantial attention. Process data reflect how students approach a task (i.e., question or item) and arrive at their given response. For a game/scenario-based task, a student was required to complete a sequence of actions in order to reach a solution to a problem. The analysis of such process data may help refine the associated scoring rubrics (Hao et al., 2015). An important aspect on process data is *response time* (von Davier et al., 2019). In a computer platform, a log file stores time-stamped action sequences to facilitate later analysis; in this case, each student’s activities about what happened and when.

In a writing assessment setting, keystroke log data can capture students’ behavior as they respond to writing assignment prompts (Chukharev-Hudilainen, 2014), far more so than the traditional (pencil–paper based) assessment. Traditional measurements of writing competency are reflected in a single score provided by a human rater (e.g., a teacher). This holistic score is a measurement of the final *writing product* (Correnti et al., 2012). So as to better understand the *writing process*, modeling keystroke log data may reveal unique patterns that are meaningful (Wengelin et al., 2009). This evaluative method may eventually help establish a writing behavior profile for each student. The hope is that such profiles consist of information collected from both the product and process of writing, together representing a student’s learning status (Xu and Qi, 2017), and will contain richer information to further influence the ongoing or future writing instruction (Baaijen and Galbraith, 2018).

A primary challenge in analyzing keystroke log data is centered around extracting meaningful features or variables. Most of the relevant studies have employed descriptive statistics or data reduction techniques. For example, Conijn et al. (2019) found that properties including a written essay’s length, the total time spent on completing the essay, and the frequency of revision, differed across distinct writing tasks. Medimorec and Risko (2017) examined pause data and found meaningful differences among the pause frequencies at different text boundaries (e.g., between sentences); specifically, the registered number of pauses at word boundaries (e.g., between words) was higher for argumentative essays than for narrative essays.

As for data reduction techniques, Zhang and Deane (2015) defined nearly 30 features by distinguishing (1) locations (e.g., pause within words), from (2) actions (e.g., deletion). Employing principal component analysis (PCA), they found four underlying factors of the writing process that are measurable with keystroke log data: (1) general fluency; (2) phrasal and chunk-level editing; (3) local editing; and (4) planning and deliberation. Similarly, Baaijen et al. (2012) defined 16 types of pauses and revisions. They also used PCA to identify the underlying dimensionality of the process data.

In addition, some studies explicitly stated that they adopted machine learning techniques to analyze log data. Uto et al. (2020) used a time- and learner-dependent hidden Markov model to analyze the writing process. Sinharay et al. (2019) used a regression tree to identify the association between writing process features and writing product features. Using a set of process variables, Sinharay et al. found that their machine learning technique slightly outperformed linear regression in predicting essay scores. In line with the current research trends in log data analysis, they pointed out the need of a variety of methods to “help explain the complex writing processes and validate the variables extracted from the writing processes for educational purpose” (p. 134).

Thus, in this research, we aim to enrich the log data analysis literature by providing analysis on a large-scale digital data collected from a writing assessment. After examining the literature, we noted that distribution-based analysis is lacking. The advantage of researching the distributions of response time (e.g., pause data) is notable; that is, the parametric methods can provide meaningful summary statistics to represent each student. However, thus far, the only work incorporating a distribution-based analysis in large-scale log data was conducted by Guo et al. (2018). While their work was a promising effort to quantify the writing process, the results were ambiguous, in terms of the meaning of the parameters extracted.

In this research, we add value to the extant literature by researching the probability distribution of log data as well as highlighting the cognitive basis of process data. We endorse the idea that the cognitive models should be the foundation when analyzing log data; any identified features or unique patterns can thus be tied back to the cognitive model, for the sake of interpretability. Cognitive science and time data have been examined together through distribution analysis, probably because distribution-based measures can help determine the characteristics of indirect evidence about latent processes. Alternatively, the Gaussian distribution is less useful to model human response time. White and Staub (2012) studied the distribution of fixation durations during reading using an ex-Gaussian distribution. Zhang et al. (2018) explored response delay in terms of attention, by comparing individuals on the parameters extracted from the Gamma distribution. Palmer et al. (2011) employed a set of distributions including ex-Wald and Weibull distributions to interpret the mechanism in *visual search*. In the brief review below, we describe the cognitive model of writing and the related empirical evidence.

### Cognitive Model of Writing and Empirical Evidence

Researchers have adopted perspectives rooted in the literature on the cognitive allocation of writing, which offers a refined account of how students allocate their cognitive resources during time spent writing (Graham and Perin, 2007). A particular focus is on how the writing process is a problem-solving process (Peskin and Ellenbogen, 2019). Writing requires students to solve a series of rhetorical, conceptual, and linguistic problems, and writers must allocate their cognitive resources in a goal-directed process (Wengelin et al., 2009). They may draw on knowledge stored in long-term memory, or create an image for the audience (Kellogg, 1996). In the oft-utilized model proposed by Flower and Hayes (1981), for instance, three main cognitive processes occur (in any order) during text production: planning, translating, and reviewing. The *planning* involves idea generation; *translating* is about forming a tentative text by elaborating the conceptual structure from the previous stage; and *reviewing* is related to making changes to the text, and to making comparisons between the written text and the intended text in one’s mental depiction. MacArthur and Graham (2016) further summarized these cognitive activities as the following: “These include planning what to say and how to say it, translating plans into written text, and reviewing to improve existing text. The use of these cognitive processes is thought to be under the writer’s direct control (managed by a control process referred to as the Monitor)” (p. 26).

Indeed, writing demands multiple fine-grained mental operations that interact recursively; it is not merely an effortless routine performed in linear fashion. As McCutchen (2000) described, writing involves the coordinated use of mental operations, including reflection and text interpretation. Transcribing words onto the page is related to orthographic skill (Berninger, 1999). Young learners (or less skilled writers) may struggle to learn other (higher) skills, such as sentence planning or text structure developing, if they have low competency in the basic ones (Graham et al., 2012). Along this line, Deane et al. (2011), Deane, 2014 proposed a multi-layer cognitive model of writing. The model specified a set of layers in the low-to-high cognitive processes. For example, lexical/orthographic skill is part of a lower cognitive process. whereas verbal/textual skill is thought to reflect higher cognitive processes.

As mentioned above, cognitive models have granted a set of possible theoretical rationales to the writing process. Within this scope, historically, researchers employed a variety of methods to gather empirical evidence. By using retrospective interviews, video observations, or think-aloud protocols, researchers observed students’ hand movements or collect verbal data. They suggested that time-related efficiency during text production can convey information about the patterns of writing process. Matsuhashi (1981) found that writing for different purposes (reporting, persuasion, and generalization) involved different pause patterns. Furthermore, skilled writers showed different patterns than less-skilled writers in their use of time (Flower and Hayes, 1981).

Nowadays, in a computer-based assessment, researchers have focused on the mouse click and type of every key press and key release. Parsing methods include (1) *Inputlog* (Leijten and van Waes, 2013); (2) *Scriptlog* (Strömqvist and Karlsson, 2002); and (3) *Translog* (Jakobsen, 2006) can thus capture the writing process. These methods rely on the same core technique, that is, identifying character input and recording the length of time (measured by millisecond) between inputs. Subsequently, with the parsed log data at hand, researchers can then further analyze the writing process.

## The Present Research

### Research Goal

The goal for this research is to identify unique patterns in pause data. We will not be able to fully address a topic this large in a single study. Therefore, with reasonable precision on the model estimation, we aim to (1) illustrate how a mixture modeling approach can help capture effects that may otherwise elude detection, as well as to (2) identify whether such underlying mechanism is consistent across different writing genres. The remainder of the paper is structured as follows. Firstly, we describe the research context and the preliminary analysis. Secondly, we introduce the large-scale data used in this research. Thirdly, we describe the procedures for modeling the data. Finally, we present the results, in terms of new patterns found in pause data.

### Research Context

The *Cognitively Based Assessment of, for and as Learning* (CBAL) Writing assessment is a research initiative developed at Educational Testing Service (ETS). As a computer-based assessment in K-12 education, the CBAL Writing assessment intends to measure literacy skills (reading, writing, and thinking) collectively (Bennett et al., 2011; Bennett et al., 2016). It requires students to solve a series of rhetorical, conceptual, and linguistic problems (Deane et al., 2008), posed following a reading passage presented to students. The assessment items consist of a series of multiple-choice questions, short open-ended questions, and essay writing prompts. The scoring for the essay writing tasks was originally based on three strands: Strand I – *sentence-level control*; Strand II – *document-level control*; and Strand III – *critical thinking*. (In later work, Strand I and Strand II were combined). Thus, human rater scoring rubric contains two sorts of scores: Strand I and Strand III.

Multiple pilot studies for the CBAL Writing assessment were conducted between 2007 and 2009, with four writing genres included in the writing tasks: (1) persuasive writing; (2) literary analysis writing; (3) argumentation and summarization writing; and (4) informational writing. Four writing prompts were included – their relevant codewords are, respectively, *ServiceLearning*, *InvasivePlantSpecies*, *BanAds*, and *MangoStreet.* Students wrote about one of these topics within a 45-min span.

After students’ responses were collected, Almond et al. (2012) used the pilot study data to conduct a preliminary analysis, where the sample size was 68 student essays. They developed a data parsing engine to classify the log data gathered. They also identified significant locations of writing pauses according to 8 linguistic contexts: *WithinWord*, *BackSpace*, *BetweenWord*, *BetweenSentence*, *BetweenParagraph*, *MultipleBackspace*, *SingleBackspace*, and *Edit*. These linguistic contexts have categorized (1) the action (e.g., *Edit*), or (2) pause location (e.g., *WithinWord*) of a large stream of information. The definition of *WithinWord*, for instance, is “[the] writer is pausing within a word.” The definition of *Edit*, on the other hand, describes a scenario in which “[the] writer is pausing before cut, paste, or replace operations or before using a mouse to navigate to a different part of the essay” (see details on page 6).

Furthermore, Almond et al. found some pause events occurred less often; that is, the number of observed pause events in that linguistic context was small. For example, the pause events between paragraphs (i.e., *BetweenParagraph*) were rarely captured by the data parsing engine, because most students only wrote 2–3 paragraphs. Thus, these authors have suggested that future analysis should focus on common pause events such as *WithinWord*. In addition, they found that the distribution of pause events on log scale for a linguistic context (e.g., *BetweenWord*) was highly leptokurtic. A mixture of lognormal distributions could explain such high kurtosis; a typical mixture model has the shape of (1) heavy tail or (2) high kurtosis. However, the sample of 68 essays was not sufficiently large to offer a full evaluation on the model-data fit, nor to identify new patterns emerging in the pause data.

### Data

In this research, we will analyze a sample of students who were in grade 8 and the data were collected across 24 schools in 18 U.S. states. Although the participating schools were volunteers, the data collection process included attention to producing a sample (e.g., high vs. low minority enrollment, high vs. low poverty schools) that was balanced demographically (Fu et al., 2012). We examine the sample of 1,054 essays in two writing genres (shown in Table 1):

**TABLE 1:** Datasets analyzed in this research.

Genre A: Literary writing	Genre B: Argumentative writing
Writing prompt: MangoStreet	Writing prompt: BanAds
Dataset 1: WithinWord (*N* = 1,054)	Dataset 2: WithinWord (*N* = 1,054)

(1) argumentation writing (*BanAds* prompt): Students wrote about whether there should be a ban on television advertisements aimed at children under age 12.
(2) literary writing (*MangoStreet* prompt): Students reviewed three excerpts from the novel *The House on Mango Street*, then wrote about it.

In each dataset, the number of pause events varied from essay to essay. Some essays are short, that is, they contain a small number of pause events. Because shorter essays produce very minimal information. in this step, we conducted data cleaning. When an essay met the following two criteria at the same time, then the essay was deleted:

(1) contained fewer than 30 pause events, and
(2) received human scores (i.e., Strand score I and Strand score III) of 0.

After data cleaning, the final sample sizes used in this research are listed in Table 2. In the analysis of the writing prompt *BandAds*, the sample size is 963, respectively. In the analysis of writing prompt *MangoStreet*, the sample size is 981. Because each essay contains different numbers of pause events, the descriptive statistics can thus show how the number of pause events is distributed across essays (see Table 2).

**TABLE 2:** Descriptive statistics for the distribution of essay length (after data cleaning).

	BanAds	MangoStreet

	*N* = 963	*N* = 981
Minimum	31.0	31.0
The 1st quartile	281.0	186.0
Median	532.0	364.0
Mean	567.8	443.9
The 3rd quartile	787.0	633.0
Maximum	2, 351.0	1, 652.0

After data cleaning, we calculated the ratio between the mean of numbers of characters typed across these essays and the mean of numbers of pause events produced across these essays. For BanAds prompt, 91 were deleted. For the remaining 963 essays, the magnitude of the ratio is 1.69. For the deleted 91 essays, we calculated the same index, the associated magnitude is 20.92. This implies that the deleted data is not a representative random sample to the whole dataset. For the deleted data, larger ratio (20.92) indicates the denominator is much smaller than the numerator, namely, the mean of numbers of pause events is much smaller than the mean of numbers of characters typed. This is understandable, because for most deleted cases, the number of observed pause events is small, even as small as 0. Students typed one word (around 4–5 characters) then submitted the final writing product where 0 pause event was captured by the keystroke log engine. The similar pattern was found in *MangoStreet* prompt.

### Finite Mixture Model

Finite mixture models are useful when observations are taken from complex heterogeneous data, which occurs when consequential subpopulations exist within an overall population. Each of these subpopulations is a mixture component in a mixture model; in other words, a random variable is drawn from a distribution which consists of *K* components (McLachlan and Peel, 2000). The binary indicator, *k*, is the index for the mixture component, which can assume a value between 1 and *K* (where *1 ≤ k ≤ K*). The density function of the mixture distribution is given by equation (1) where *g* (*x*|θ) indicates that the random vector *x* is distributed according to the *k*th mixture component, with the component parameter θ_*k*_. The mixing proportion parameter is π_*k*_, the proportion of the population from the mixture component *k*. The constraint of π_*k*_ is $\sum_{k=1}^{K}\pi_{k}=1.$

(1) $$g\,\left(y|\theta \right)=\sum_{k=1}^{K}f\,\left(y|\theta_{k}\right)\cdot \pi_{k}$$

**FIGURE 1:**
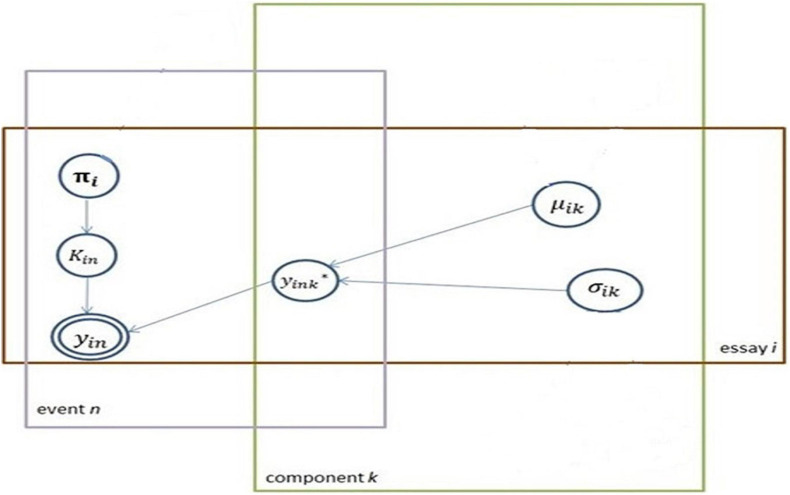
The proposed model in plate notation.

Computationally, finite mixture models are not identifiable without imposing additional restrictions. Thus, the substantive knowledge is applied only whenever the finite mixture model is used in a certain context. In this research, the component with the smaller value for the mean is arbitrarily defined as the low-cognitive component. Usually, mixture components are assumed to follow the same parametric family (e.g., all Normals, all Poissons) with different parameter vectors. In this research setting, all mixture components are Normals, with a set of means and standard deviations (SDs) to be estimated (μ_*k*_andσ_*k*_). Each essay *i* is indexed with the component term. A mixture model is applied to each essay. A set of parameters thus is estimated for each essay. The proposed modeling approach is illustrated with a plate notation, shown in Figure 1.

In the mixture of lognormal models, random variables – pause events-are transformed onto the log scale, *x* = log(*y*). The density function, with the mean and the SD for each component and mixing proportion, is given in Equation (2).

(2) $$g\,\left(x|\theta \right)=\sum_{k=1}^{K}f\,\left(x|\theta_{k}\right)\cdot \pi_{k}$$

In practice, it is very common for the component membership to be unknown. In this research context, a ramification is that it is unknown from which cognitive process the observed pause event is drawn. Associated with each pause event, there is a term, the *latent mixture indicator* **z** where **z**∈(1,2,…, *K*), is a label variable attached to each observation. Each observation comes from exactly one component (or, one cognitive process) where *P*(*z* = *k*) = π_*k*_ for *k* = 1,2,…,*K*. Thus, equation 2 can be re-expressed as:

$$g\left(x|\theta ,z=k\right)=f\left(x|\theta_{k}\right),P\left(z=k\right)=\pi_{k}$$

Another way to describe the latent indicator variable is that observed pause events *x* are incomplete data unless the associated cognitive process labels are given. The complete-dataset can then be defined as: *c* = (*x*,*z*). The complete data density is denoted as *h*_θ_.

In terms of computing, the most common algorithm is an expectation–maximization (EM) algorithm for estimating finite mixture model (Dempster et al., 1977; McLachlan and Peel, 2000). The EM algorithm maximizes the operator *Q*, as following, given in Equation (3):

(3) $$Q\left(\theta |\theta^{\left(t\right)}\right)=E\left[logh_{\theta }\left(C\right)|x,\theta^{\left(t\right)}\right]$$

The θ^(*t*)^ is the current value at iteration *t*. The iteration θ^(*t*)^→θ^(*t* + 1)^ is set up by E-step and M-step:

(1) E-step: compute *Q*(θ|θ^(*t*)^)
(2) M-step: set θ^(*t* + 1)^ argmax *Q*(θ|θ^(*t*)^)

## Results

### Mixture Parameters

The previous pilot study (*N* = 68 essays) was not able to fit the data with a variety of mixture distributions. In this research, that task is accomplished. The parameter estimation throughout this research was conducted in the R environment (R Core Team, 2013), using the *mixtools* package (Benaglia et al., 2009, 2021). Specifically, a mixture of gamma distribution and a mixture of exponential distribution were used. These models, as presented in the literature, were used to model highly skewed data, including human response time data. Since none of these models has converged, the estimation results are not provided in detail. The only model converged was the mixture of lognormal models (described above). In this section, the relevant results are provided.

**FIGURE 2:**
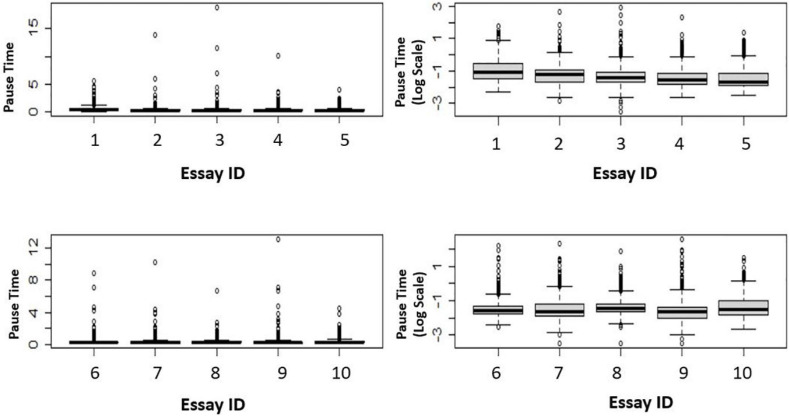
Box plots of 10 essays.

Firstly, to examine the distributions, we present a set of box plots. For illustration purposes, ten *BanAds* essays were randomly selected. Figure 2 shows that even after transforming the data onto the log scale, the data still had high kurtosis.

Secondly, a set of lognormal mixture models are used to fit the data where a fixed value for *k* (i.e., *k* = 2, 3, 4, 5) is applied, in this research. Namely, the two-component mixture of lognormal model, the three-component mixture of lognormal model, the four-component mixture of lognormal model, and the five-component mixture of lognormal model. Table 3 shows the number of parameters estimated in each model. These models are applied to each essay.

**TABLE 3:** The number of parameters estimated in each model.

Model	*k* = 2	*k* = 3	*k* = 4	*k* = 5
Number of parameters	5	8	11	14
Parameters	μ_1_,σ_1_, π_1_, μ_*2*_, σ_2_ (π_*2*_ = 1-π_*1*_)	μ_*1*_,σ_*1*_,π_*1*_ μ_2_,σ_2_,π_*2*_, μ_*3*_, σ_*3*_ (π_*3*_ = 1-π_*1*_-π_*2*_)	μ_*1*_, σ_*1*_, π_*1*_, μ_*2*_, σ_*2*_, π_*2*_, μ_*3*_, σ_*3*_, π_*3*_,μ_*4*_, σ_*4*_ (π_*4*_ = 1-π_*1*_-π_*2*_-π_*3*_)	μ_*1*_, σ_*1*_, π_*1*_, μ_*2*_, σ_*2*_, π_*2*_, μ_*3*_,σ_*3*_, π_*3*_, μ_*4*_, σ_*4*_, π_*4*_,μ_*5*_, σ_*5*_ (π_*5*_ = 1-π_*1*_-π_*2*_-π_*3*_-π_*4*_)

After counting the number of essays converged to these four models, (AIC, Akaike, 1974) and Bayesian information criterion (BIC) statistics are calculated using Equations 4, 5. The notation *N* indicates the number of pause events for a given essay, and the notation *p* refers to the number of parameters estimated in that model. (AIC and BIC statistics for mixture models’ data-model fitting are not available in the *mixtools* package. The loglikelihood values *are* available in the package). Most essays converged to the mixture models. Table 4 shows the convergence results across two writing genres.

**TABLE 4:** Summary of model convergence results.

The number of essays	BanAds	MangoStreet
Analyzed in total	963	981
Converged to the two-component model	961	980
Converged to the two and three-component models	948	967
Converged to the two-, three-, and four-component models	927	903
Converged to the two-, three-, four-, and five-component models	886	900

(4) A⁢I⁢C=-2×L⁢o⁢g⁢l⁢i⁢k⁢e⁢l⁢i⁢h⁢o⁢o⁢d+2×p.

(5) B⁢I⁢C=-2×L⁢o⁢g⁢l⁢i⁢k⁢e⁢l⁢i⁢h⁢o⁢o⁢d+p×l⁢o⁢g⁢(N)

With respect to the *BanAds* writing prompt, a total of 886 essays converged to all the two-, three-, four-, and five-component mixture of lognormal models. For *MangoStreet*, the total of 900 essays converged to the two-, three-, four-, and five-component mixture of lognormal models. The model selection criterion for AIC and BIC is that: the lower value the AIC or BIC has, the better the model it is. In this research, the AIC statistics and BIC statistics are consistent when selecting models, meaning that AIC and BIC never “disagree” with each other. Put another way, it is never the case that AIC favors one model, but BIC favors another model. Table 5 shows the number of essays is selected by the AIC and BIC, for each particular model.

**TABLE 5:** Model selection results.

AIC and BIC	BanAds	MangoStreet
Favored for the two-component model	24	39
Favored for the three-component model	431	409
Favored for the four-component model	410	398
Favored for the five-component model	41	54

According to Table 5, it appears that the three- and four-component models are competing models. To further identify the number of mixture components, the distance between the components’ means is also considered. Typically, when the number of components is increased in a mixture model, the model-data fit tends to be improved, as reflected by AIC or BIC statistics. However, if any of two components is not well separated, the precision of estimated parameters will be low. Merely increasing the number of parameters to be estimated will lead the over-fitting issue, so it is best to compute the magnitude representing how far two components are from each other. The distance for a given pair of means was calculated for the three-component mixture model and then for the four-component mixture model. The three-component model produced three quantities to represent the distance between means. Quantity 1 is the distance between μ_1_ and μ_2_; Quantity 2 is the distance between μ_1_ and μ_3_; and Quantity 3 is the distance between μ_2_ and μ_3_ (shown in Table 3). Similarly, the four-component model produced six quantities to represent the distance between means.

The cut-point of 1 and the cut-point of 0.3 are applied to the three-component model and to the four-component model. For each model, if the distance between two means is equal or larger than 1, it indicates two components are well separated. If the distance is equal or larger than 0.3, it indicates two components are reasonably separated. After calculating any possible distance for each model, the percentage of well-separated cases or reasonably separated cases can then be calculated. For illustration purpose, Table 6 shows the results of the *BanAds* prompt. The same pattern was found in *MangoStreet* prompt.

**TABLE 6:** The distance of any of two components’ means.

	Three-component model (%)	Four-component model (%)
Well-separated components	6	0.9
Reasonably separated components	72	47

The larger model (the four-component mixture of lognormal model) has too little separation. The magnitude of distance between two components tends to be low across all the possible pairs of components, which may reflect the low precision of estimation. This implies that using the four-component mixture of lognormal model should be with caution in this research context.

### Correlation Analysis

In addition, we conducted a series of correlation analysis for a set of variables: (1) human scores, and (2) the mixture parameters. Human scores refer to Strand I and Strand III scores, which reflected the quality of each final writing product. The mixture parameters refer to the estimated mean, SD, and the mixing proportion for each essay. Different mixture model produced different number of parameters, as shown in Table 3. For illustration purposes, we select one analysis to report, see below: the parameters from the three-component mixture modeling for the *BanAds* writing prompt. Eight parameters were produced by each essay: mean and SD for low-cognitive component (μ_1_,σ_1_); mean and SD for medium-cognitive component (μ_2_,σ_2_); mean and SD for high-cognitive component (μ_3_,σ_3_); and two mixing proportions (π_1_, π_2_). The third mixing proportion is not estimated because, by definition,π_*3*_ = 1−π_*1*_−π_*2*_ (shown in Table 3).

Table 7 indicates that there is a correlation between the proportion of pause events in the low-cognitive component (π_1_) and the two human scores (Strand score I and Strand score III). The index is statistically significant, although the magnitude is low. This implies that students who spend more time on the low-level cognitive processes tend to have lower strand scores. The confidence interval is presented in parentheses. Same patterns are found in the other models (i.e., the two-, the four-, and the five-component models), namely, the strongest correlation between the human scores and the mixing proportion parameter of the low-cognitive component is found.

**TABLE 7:** Correlation between the human scores and the mixture parameters.

	Parameter	Strand	Strand
		score I	score III
θ_*1*_	μ_1_	*−*0.27* (*−*0.33, *−*0.21)	*−*0.24* (*−*0.30, *−*0.18)
	π_1_	*−*0.23* (*−*0.29, *−*0.17)	*−*0.20 (*−*0.26, *−*0.14)
	σ_1_	*−*0.20 (*−*0.26, *−*0.14)	*−*0.22 (*−*0.28, *−*0.16)
θ_*2*_	μ_2_	*−*0.19 (*−*0.25, *−*0.13)	*−*0.16 (*−*0.22, *−*0.10)
	π_2_	*−*0.19* (*−*0.27, *−*0.15)	0.24 (0.18, 0.30)
	σ_2_	*−*0.12 (*−*0.18, *−*0.06)	*−*0.10 (*−*0.16, *−*0.04)
θ_*3*_	μ_3_	0.06 (0, 0.12)	0.05 (*−*0.01, 0.11)
	π_3_	0.09 (0.03, 0.15)	0.12 (0.06, 0.18)
	σ_3_	*−*0.10 (*−*0.04, *−*0.16)	*−*0.11 (*−*0.17, *−*0.05)

θ_1_ is the parameter of the low cognitive component; θ_2_ is the parameter of the medium cognitive component; θ_3_ is the parameter of the high cognitive component.*p < 0.05.

**FIGURE 3:**
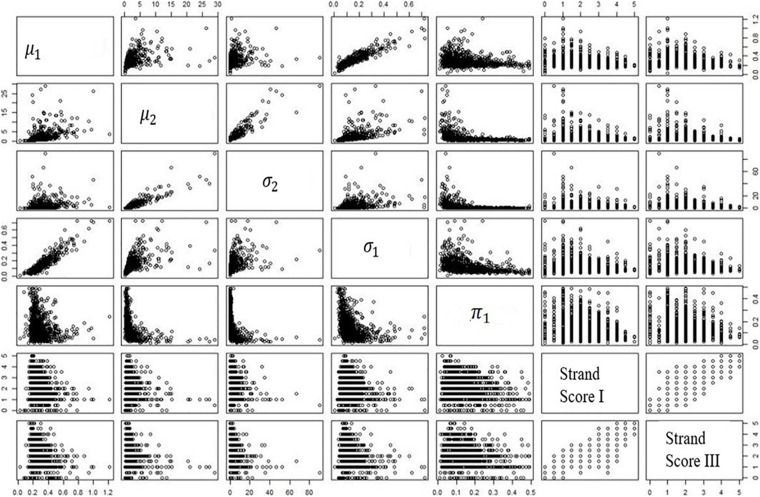
Correlations among human scores and mixture parameters.

Table 7 is the numeric expression of correlations; Figure 3 shows the correlation plots among variables. Interestingly, a complex relationship between the mixing proportion parameter and the two sets of human scores is found in the plot.

Figure 3 shows that the correlation is stronger at the upper bound of the plot. In other words, those students with low human scores have a wider range of values across the mixing proportion parameter; the students with higher human scores do not possess such a wide range of values in the mixing proportion parameter. This finding is consistent across two writing genres. Among all the estimated parameters, the mixing proportion on the low cognitive process plays the primary role. All the patterns mentioned above are found in *MangoStreet* writing prompt.

## Discussion and Conclusion

In this section, we connect our findings to the existing literature. For each essay under examination, we applied a set of mixture models: the mixture of gammas, the mixture of exponentials, and mixture of lognormals. The model convergence results showed that the mixture of lognormals had desirable results. Subsequently, we determined the number of mixture components (*k*) for the lognormal model. As the AIC and BIC statistics showed, the three-component and the four-component models were similar in terms of model-data fit. In the computational statistics literature, the choice of *k* is a long-standing topic of discussion (Fowlkes, 1979; Seo and Lindsay, 2010; Huang et al., 2017), given that “estimating *k* can be difficult in practice and often one prefers to choose a large *k*, with the risk that the *true distribution* has fewer components” (Rousseau and Mengersen, 2011, p. 690). The concern is that increasing the number of parameters leads to overfitting, with low precision on the estimations.

In order to further determine the choice of *k*, we calculated the distance between a pair of component means for the three-component model and for the four-component model. As shown in Table 6, the components are very close in the four-component model, which suggest that the precision of estimation is low. Thus, we ultimately chose the three-component model in this research. In the case that two components are very close, their precision can be increased only when the sample size is very large, in this case, meaning that the number of pause events per essay has to be large enough in order to reach the certain precision level.

As Chen et al. (2008) have described, “when the component densities are not well separated, much larger sample sizes are needed to achieve precision similar to that in our simulation” (p. 459). In their study, Chen et al. used the sample sizes of 100, 500, and 2,500. In this research, the sample sizes varied from essay to essay; as shown in Table 2, the sample sizes for most essays were not able to achieve the desirable precision. Thus, the four-component model is not chosen, due to the high risk of reduced precision. As for how close is close in terms of component means, in this research, we used the cut-points *d* = 1 and 0.3. In the simulation study conducted by Chen et al. (2008), they set the distance between the means of two component as *d* = 3. In the classic study conducted by Redner and Walker (1984), they set *d* = 1,2, …, 6 in their simulation study.

We chose to use the three-component model, a decision which did not merely rely on the statistical properties mentioned above (e.g., model selection index, the precision of estimation), but also took the research setting and cognitive foundation into account. As specified earlier, students had 45 minutes to complete the writing task. Therefore, when a mixture modeling is applied in this testing occasion, the number of mixture components may be limited by the brevity of the task, meaning that some comparatively rare components might not appear. This implies that higher-level cognitive processes may not appear in the short time span under investigation.

Thus, in terms of generalizability, the explanations we suggested should be further evaluated at various writing conditions or using different samples. This does not present difficult work to be done. In the existing literature, researchers have explicitly stated that differences in writing conditions and samples should be evaluated. Malekian et al. (2019) examined the log data collected from 107 college students, where the writing task is a 1,000-word essay and the writing score is included in students’ final course grade. This sort of testing condition may offer more pause events to model higher-level mixture components.

Furthermore, the mixture modeling produced a set of parameters for each essay. These parameters were used as writing process features. Among all the estimated parameters, the mixing proportion on the lower-level cognitive processes plays the primary role. As shown in Table 7, the correlations related to the lower-level cognitive process component could tentatively be interpreted as the *fluency effect*, and modeling the *WithinWord* linguistic context data might reflect a measure of dysfluency with orthography. Orthography is the least demanding of the measured cognitive processes (Deane et al., 2008; Deane et al., 2011; Conijn et al., 2019). If an 8^*th*^ grade student has too many pauses at the location of typing a word (*WithinWord* pause data), it is more likely that he/she has difficulty spelling rather than that he/she is instead engaging with a higher-level cognitive process (e.g., word choice or intention reflection). As described by McCutchen (2000), writing fluency is a paramount consideration because inefficient low-level cognitive processes may impose on working memory resources, which in turn hinders deeper engagement in skills such as sentence planning or intention reflection. In addition, the fluency effect in this research is consistent across writing genres. It echoes back to Conijn et al.’s (2019) finding, that is, the features, particularly for within words, are robust across writing tasks.

In terms of the low magnitudes between mixture modeling parameters and the human scores, this is similar to Guo et al.’s (2018) finding where they used estimated stable distribution parameters to correlate with human scores. The low magnitudes on the correlations are not surprising, because the goal of the present research is not about replacing human score with the extracted process features. Instead, the goal is to gain knowledge about how students write, because writing instruction has always been centered around *writing process*.

In this research, the students knew that the scores from the CBAL Writing would not affect their grades; that is, the assessment was a low-stake test. Therefore, some of students failed to make serious effort. Whenever the number of pause events in an essay was less than 30 and the human scores for this essay were 0, the essay was not included in the analysis. In total, around 100 essays per writing genre were excluded on the basis of these criteria. Among all the essays, about 9% of the essays were deleted. The main reason for deleting them is that a small number of pause events (i.e., less than 30) per essay would produce extremely low precision on parameter estimation. As pointed out earlier in this paper, the goal of this research was to gain a reasonable precision on parameter estimation. With too few observations together with too many parameters, the estimation results would not possess the desirable statistical stabilities. As shown in Table 3, for the two-component model, five parameters needed to be estimated; for the three-component counterpart, eight parameters; for the four-component counterpart, 11 parameters; for the five-component counterpart, 14 parameters. Also, for these deleted essays, the fact that human scores of 0 for them is not able to contribute to the subsequently correlation analysis conducted in this research.

After data deleting, we calculated the ratio between the mean of numbers of characters typed across these essays and the mean of numbers of pause events produced across these essays. The magnitude of the ratio implies that the deleted data is not a representative random sample to the whole dataset. In practice, it is common that students did not exert serious effort on the low-stake tests. In assessment literature, if response times are available from computer platforms, a set of test-taking behaviors such as rapid guessing behavior (in which a student will complete a whole test or an item within a very short time), or producing a minimal number of pause behaviors, can be further investigated. A large body of methodological work (e.g., Kong et al., 2007; Lee and Jia, 2014) has examined the issues about students’ *test-taking engagement*. In future, detailed research should be conducted to examine the students’ characteristics on test-taking engagement between the deleted essay (students) and the remaining ones.

In summary, this research shows that the mixture of lognormal models captures important characteristics of the writing process not captured by other methods in the existing literature. For some students (essays), the model selection criterion (i.e., AIC and BIC) favored one model; for others, the criterion favored another model. Future studies may examine whether a systematic difference exists between students whose results are best explained by one among the two-, three-, four-, and five-component models. Moreover, attention should be paid to the fact that students with low human scores have a wide range of values on the mixing parameter, but students with higher scores do not have the wide range of values on the mixing parameter. Future studies may therefore examine whether this indicates a systematic difference between these groups.

This research modeled the observed pause events gathered by the keystroke logs so the extracted features become meaningful and interpretable through the low-to-high-level cognitive processes. As elaborated throughout the study, the underlying assumptions include (1) that each observed pause event at any location is a random variable drawn from one of the cognitive processes; (2) different cognitive process takes different amount of time; and (3) some pause locations are more frequent than others.

In the log data analysis community, researchers have been seeking mechanisms to explain and quantify students’ writing process. Currently, studies capturing the dynamics of text production have advanced only far enough to bring possibilities to light. This present research is an early-stage attempt at an effort to tie mixture model parameters to the multi-layer cognitive model of writing. This research, therefore, aligns with the scholarly community in search of a greater understanding of writing behaviors.

## Data Availability Statement

The data analyzed in this study is subject to the following licenses/restrictions: the data that support the findings of this study are available from Educational Testing Service. Requests to access these datasets should be directed to https://www.ets.org/.

## Ethics Statement

The studies involving human participants were reviewed and approved by the Florida State University – Institutional Review Board (IRB). Written informed consent from the participants’ legal guardian/next of kin was not required to participate in this study in accordance with the national legislation and the institutional requirements.

## Author Contributions

The author confirms being the sole contributor of this work and has approved it for publication.

## Conflict of Interest

The author declares that the research was conducted in the absence of any commercial or financial relationships that could be construed as a potential conflict of interest.

## Publisher’s Note

All claims expressed in this article are solely those of the authors and do not necessarily represent those of their affiliated organizations, or those of the publisher, the editors and the reviewers. Any product that may be evaluated in this article, or claim that may be made by its manufacturer, is not guaranteed or endorsed by the publisher.
